# Integrated risk scores from N6-methyladenosine-related lncRNAs are potential biomarkers for predicting the overall survival of bladder cancer patients

**DOI:** 10.3389/fgene.2022.906880

**Published:** 2022-08-17

**Authors:** Xin Huang, Hao-Fei Wang, Shuang Huang

**Affiliations:** ^1^ Department of Urology, Ruijin Hospital, Shanghai Jiaotong University School of Medicine, Shanghai, China; ^2^ Department of Urology, The General Hospital of the People’s Liberation Army, Beijing, China

**Keywords:** bladder cancer, N6-methyladenosine, long noncoding RNA, risk scores, prognostic signature

## Abstract

**Background:** N6-methyladenosine (m6A) is the most common form of mRNA- and long noncoding RNA (lncRNA)-specific internal modification encountered in eukaryotes, with important effects on mRNA stability, translation, and splicing. The role of m6A-modified lncRNAs (m6A-lncRNAs) in bladder cancer (BLCA) is rarely reported. This study aimed to evaluate an efficient prognostic model of BLCA in patients, based on m6A-lncRNAs, and to discover potential biological targets.

**Methods:** Differentially expressed lncRNAs were investigated in 433 BLCA samples derived from The Cancer Genome Atlas (TCGA) database. Kaplan–Meier and univariate Cox regression analyses were performed to screen for m6A-lncRNAs with prognostic roles in BLCA. We implemented Pearson correlation analysis to analyze 18 potentially prognostic lncRNAs and 20 known m6A-associated genes. Next, the data were imputed using least absolute shrinkage and selection operator (LASSO) Cox regression to establish an m6A-lncRNA prognostic signature.

**Results:** We established an integrated risk score (RS) containing five m6A-lncRNAs and constructed a nomogram that had the ability to forecast the overall survival (OS) of patients with BLCA. We showed that the predictive accuracy of the RS for BLCA prognosis was high, which was confirmed by the area under the receiver operating characteristic (ROC) curve. We analyzed the correlation between tumor immune infiltrating cells and RS in high- and low-risk patients with BLCA and used tumor immune dysfunction and exclusion to predict the effect of immunotherapy. We screened out the most relevant modules of RS through the weighted gene co-expression network analysis network and explored their potential biological functions using GO and KEGG analyses.

**Conclusion:** Our findings demonstrate that, compared with nomograms constructed using a single prognostic factor, the integrated RS represents a superior model for predicting survival in patients with BLCA, which may improve the clinical management of BLCA.

## Introduction

Bladder cancer (BLCA), also known as urothelial cancer, is the fourth most common malignancy in men and is gradually becoming more frequent in women (Lenis et al. (2020b)). BLCA is a tumor of cells that line the inside of the urinary bladder (Lenis et al., 2020a). Aggressive and invasive BLCA tumors are associated with a high degree of mortality, and molecularly diverse BLCA tumors have a high mutational rate (Jalanko et al., 2020). Therapy of invasive disease, especially with radical cystectomy and urinary diversion, has serious short-term and long-term adverse effects on the quality of life and sexual function (Lenis et al., 2020a). Advances in molecular pathology and the development of targeted therapies for BLCA have provided new insights into the complex biological processes implicated in BLCA, which may lead to more effective treatment options for a patient with this disease (Alifrangis et al., 2019; Chasov et al., 2020). Therefore, the search for therapeutic targets to treat BLCA remains a health priority.

N6-methyladenosine (m6A) is the most common internal modification of messenger RNA (mRNA) and long noncoding RNA (lncRNA) found in eukaryotes, which affects mRNA stability, translation, and splicing, and is involved in multiple biological processes (Liu et al., 2018; Yue et al., 2019). The m6A modification is a reversible process, which is mediated by the Wilms tumor 1-associated protein (WTAP) and the m6A methyltransferases methyltransferase-like 3 (METTL3) and methyltransferase-like 14 (METTL14) and is eliminated by the fat-mass and obesity-associated protein (FTO) or alkylation repair homolog protein 5 (ALKBH5) (Jia et al., 2011; Dominissini et al., 2012; Liu et al., 2014). The m6A-associated modification of RNA has been reported to play a vital role in several human cancers. For instance, METTL3 has been shown to regulate myeloid differentiation both in leukemia cells and during normal hematopoiesis (Vu et al., 2017). FTO participates in the resistance of cervical squamous cell carcinoma to chemoradiotherapy (Zhou et al., 2018). Meanwhile, ALKBH5 contributes to the oncogenicity of glioblastoma stem-like cells (Zhang et al., 2017). Furthermore, m6A modification of noncoding RNAs not only affects their transport, cleavage, stability, and degradation, but also regulates the proliferation, infiltration, and metastasis of certain tumor cells by affecting the biological function of these cells (Geula et al., 2015; Wang et al., 2018; Ma et al., 2019). To our surprise, several studies have reported that noncoding RNAs could also affect m6A modifications (Cai et al., 2018). Thus, m6A modifications of noncoding RNA and the concomitant targeting of m6A on noncoding RNAs may provide some surprising benefits for cancer therapy.

Numerous studies have now demonstrated that the expression pattern of many lncRNAs changes during the development of BLCA (Peter et al., 2014; Mao et al., 2022). Thus, specific lncRNAs are significantly associated with the prognosis of patients with BLCA and could potentially be used as predictive biomarkers for survival (Quan et al., 2018). For example, lncRNA *HOTAIR* is upregulated in BLCA and associated with poor disease-free survival (DFS), recurrence-free survival (RFS), or disease-specific survival (DSS). In contrast, lncRNA *GAS5* is downregulated in BLCA and is also associated with poor DFS, PFS, or DSS (Quan et al., 2018). lncRNA *CCAT1* has been reported to play an oncogenic role in BLCA (Zhang C. et al., 2019). However, the exact roles of m6A-modified lncRNAs (m6A-lncRNAs from here on) in BLCA remain unclear. A few studies have explored the molecular mechanisms underlying how m6A-lncRNAs contribute to the occurrence and development of BLCA, which could lead to the identification of valuable biomarkers for use as therapeutic targets in BLCA. The m6A-lncRNAs described in our study may therefore prove to be new BLCA-associated diagnostic, prognostic, and therapeutic biomarkers.

## Materials and methods

### Datasets and m6A-associated genes

We obtained The Cancer Genome Atlas (TCGA) BLCA dataset, containing data on 433 patients with BLCA and 405 corresponding clinical data records from the genomic data commons database (https://portal.gdc.cancer.gov/). TCGA patient clinical information is provided in Supplementary Table S1. In addition, referring to previous publications, the expression matrixes of 20 m6A-associated genes were also extracted from TCGA, including the expression data on readers (R) (*YTHDC1, YTHDC2, YTHDF1, YTHDF2, YTHDF3, IGF2BP1, IGF2BP2, IGF2BP3, PRRC2A*), erasers (E) (*ALKBH5* and *FTO*), and writers (W) (*METTL3, METTL14, METTL16, WTAP, RBM15, RBM15B, VIRMA, CBLLA, and ZC3H13*). For the training set for modeling, 300 cases were randomly selected and the remaining cases were used as the test set after modeling.

### Screening of lncRNAs

Based on the recognition of ensemble gene IDs, a total of 5,408 differentially expressed genes (DEGs) were identified between 433 BLCA cancer tissues and adjacent normal tissues, using the TCGA dataset together with the “limma” R package (Phipson et al., 2016) (with the criteria of |log2FC| > 1 and *p* < 0.05). Then, a total of 658 differentially expressed lncRNAs were extracted.

### Conserved motif analysis

To predict the binding sites of m6A in the lncRNAs, RMBase v2.0 (http://rna.sysu.edu.cn/rmbase/index.php) was used to compare the coding and genomic sequences of the lncRNAs. To identify the conserved motifs, the MEME program (http://meme-suite.org/) was used to determine the base content of these potential m6A binding sites on the m6A-lncRNAs.

### Bioinformatic analysis

Kaplan–Meier curves (*p* < 0.05) were generated to compare how the overall survival (OS) of patients with BLCA (derived from TCGA) is associated with lncRNA expression using the “survival” R package. Univariate Cox regression analysis (*p* < 0.01) was implemented to filter out the potentially prognostic lncRNAs. Pearson correlation analysis was initially conducted to search for m6A-lncRNAs (|Pearson R| > 0.3 and *p* < 0.001) based on 20 m6A-associated genes. According to a previous criterion (Chang et al., 2021), we randomly divided the TCGA cohort into 7 (300 cases):3(133 cases) as the training set and validation set.

Next, we performed least absolute shrinkage and selection operator (LASSO) Cox regression using the R package “glmnet” (Friedman et al., 2010) and then established a prognostic signature for BLCA patients, composed of five m6A-lncRNAs. The risk score (RS) was then calculated as follows:

$$\mathrm{Risk score}=\sum_{i=1}^{n}\mathrm{Coe}f_{i}*x_{i}$$

where $\mathrm{Coe}f_{i}$ represents the coefficients and $x_{i}\;$ refers to the FPKM value for each m6A-lncRNA. The receiver operating characteristic (ROC) curves (including 1-year, 2-year, 3-year, and 5-year survival) were drawn to verify the predictive value of the RS using the “survival ROC” package of R software. Validation sets are used to verify the generalizability of the model.

### Functional and pathway enrichment analysis

Weighted gene co-expression network analysis (WGCNA) (Langfelder and Horvath, 2008) was used to screen for gene co-expression modules that group genes with similar expression patterns.

The modules with the highest correlation levels were then used for functional and pathway enrichment analysis, which involved GO biological processes and KEGG pathways, using the “clusterProfiler” package of R software (Yu et al., 2012). Single-sample gene set enrichment analysis (ssGSEA) (Barbie et al., 2009) based on the hallmark gene set from the Molecular Signatures Database (MsigDB) was used to evaluate the tumor characteristics. Gene set enrichment analysis (GSEA) (Subramanian et al., 2005) was used to investigate potential mechanisms. All data analysis was performed using R software.

### Clinical samples collection, RNA extraction, and real-time quantitative PCR

The paired with paracancer tissue samples from 8 BLCA patients were collected from the Department of Urinary Surgery, Ruijin Hospital, Shanghai Jiaotong University School of Medicine for total RNA extraction, reverse transcription, and qRT-PCR analysis. The relative expression of the candidate genes was calculated based on the internal reference glyceraldehyde 3-phosphate dehydrogenase. The primers are listed in Supplementary Table S2. The patient clinical information is provided in Supplementary Table S3.

Total RNAs were extracted from clinical samples using Trizol reagent (15596018, Takara). Total RNAs were then reverse-transcribed into cDNA using TransScript All-in-one First-Strand cDNA Synthesis SuperMix for qPCR (AT341-01, TransGen). RT-qPCR (real-time quantitative PCR) was carried out using the PerfectStart Green (AQ601-02, TransGen) in Applied Biosystems 7,500 Real-Time PCR System.

### Statistical analyses

Kaplan–Meier curves were used to analyze the OS of patients with BLCA alongside different lncRNA expressions or RS levels, based on the expression of each of the five potentially prognostic m6A-lncRNAs selected. Univariate and multivariate Cox regression analyses were performed to assess the independent prognostic value of the m6A-lncRNAs. A nomogram was generated to show the prognostic ability of the m6A-lncRNA signature and calibration plots were constructed to validate the prognostic predictive accuracy of the nomogram. ROC curves (constructed using the “survival ROC” package of R software) and the area under the curve (AUC) were used to assess the prognostic RS-associated performance for 1/2/3/5-year OS. The statistical analyses in our study were carried out using the R programming language (version 4.0.3) and Prism 8.0 software (GraphPad, San Diego, CA, United States).

## Results

### Identification of m6A-lncRNAs in patients with bladder cancer

**FIGURE 1:**
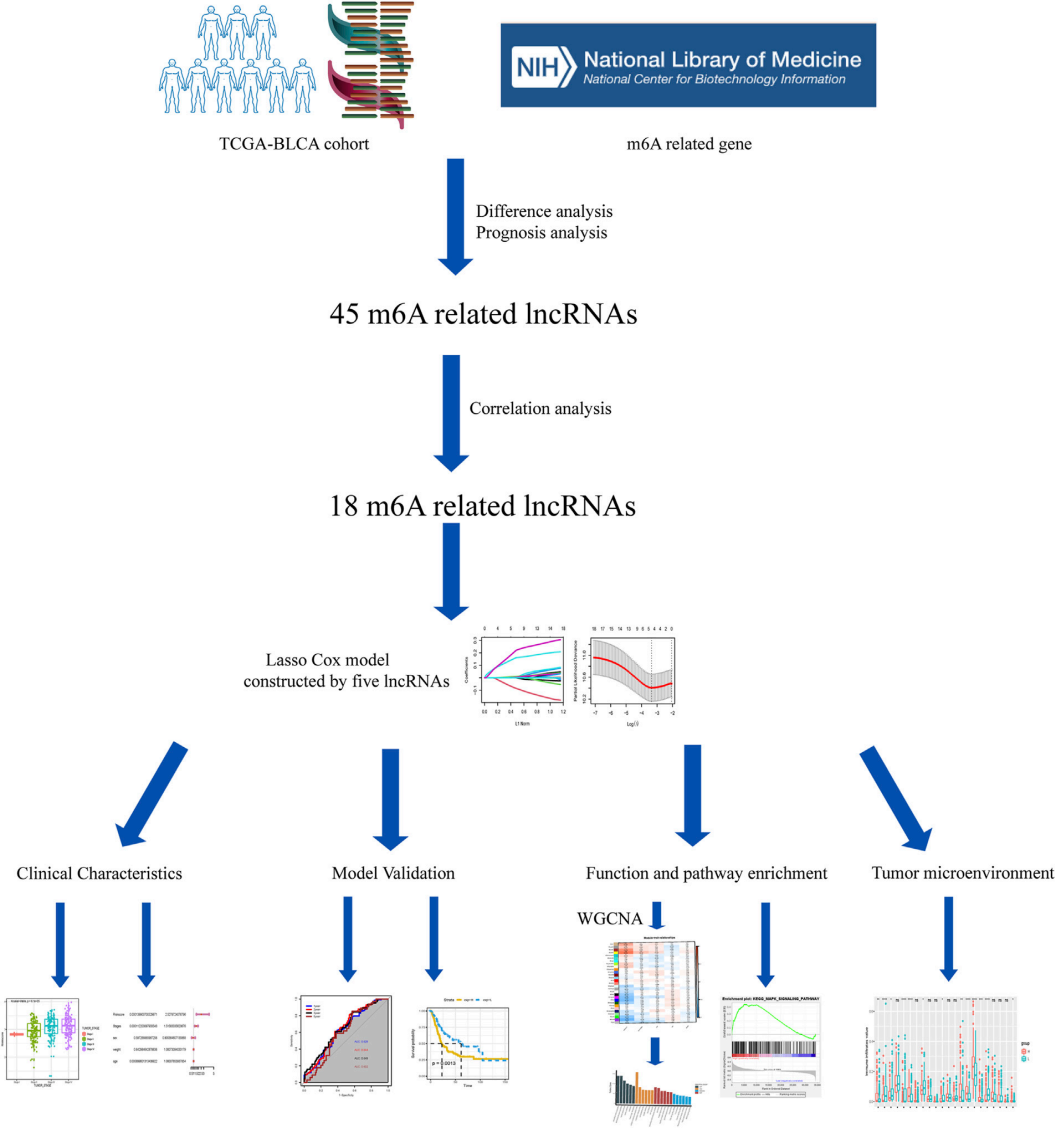
Study workflow.

**FIGURE 2:**
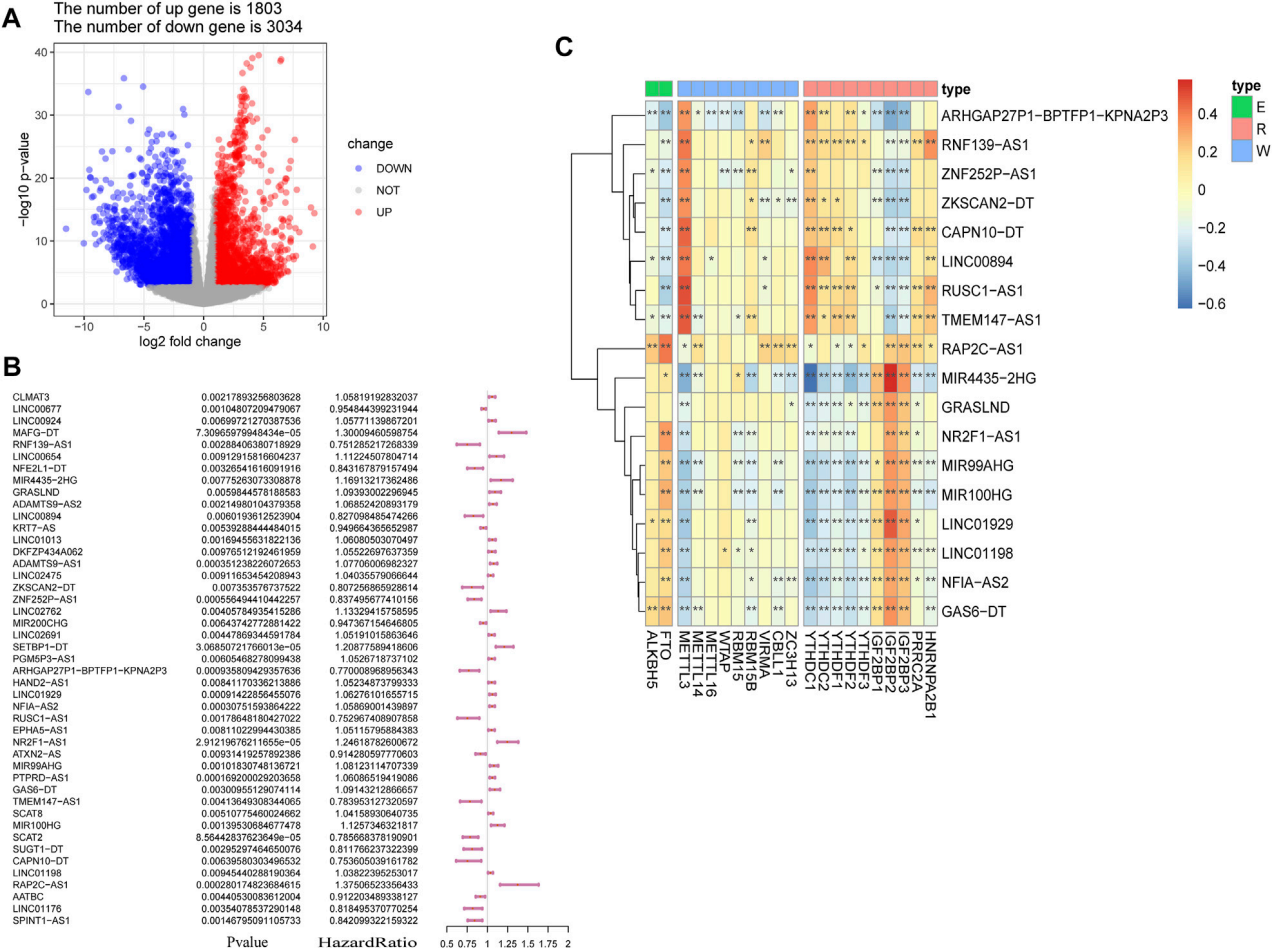
Screening of lncRNAs implicated in bladder cancer (BLCA). **(A)** volcano plot showing 4,837 DEGs between patients with BLCA and healthy controls derived from The Cancer Genome Atlas (TCGA). Blue dots represent downregulated genes; red dots refer to upregulated genes; and gray dots represent genes that were not differentially expressed. FC: fold change. **(B)** results of the univariate Cox regression analysis, performed on 45 candidate lncRNAs. **(C)** heatmap of the correlations observed between 20 m6A-associated genes and 18 prognostic m6A-lncRNAs. *p < 0.05, **p < 0.01, and ***p < 0.001.

The study workflow is shown in Figure 1. First, a total of 4,837 DEGs (1803 upregulated genes and 3,034 downregulated genes) were identified in samples obtained from patients with BLCA compared to healthy tissue samples within TCGA (Figure 2A). Then, 2,949 differentially expressed lncRNAs were selected. Combining Kaplan–Meier (*p* < 0.05) and univariate Cox regression (*p* < 0.01) analyses, we screened out a total of 45 lncRNAs, which were significantly associated with the survival rate of patients with BLCA (Figure 2B). To further refine our list of lncRNAs, from the 45 potentially prognostic lncRNAs, we selected only those that correlated with the expression of one or more of the 18 m6A-associated genes (|Pearson R| > 0.3 and *p* < 0.001) and defined these as the m6A-lncRNAs (Figure 2C).

### Construction and validation of the five m6A-lncRNA prognostic signature

**FIGURE 3:**
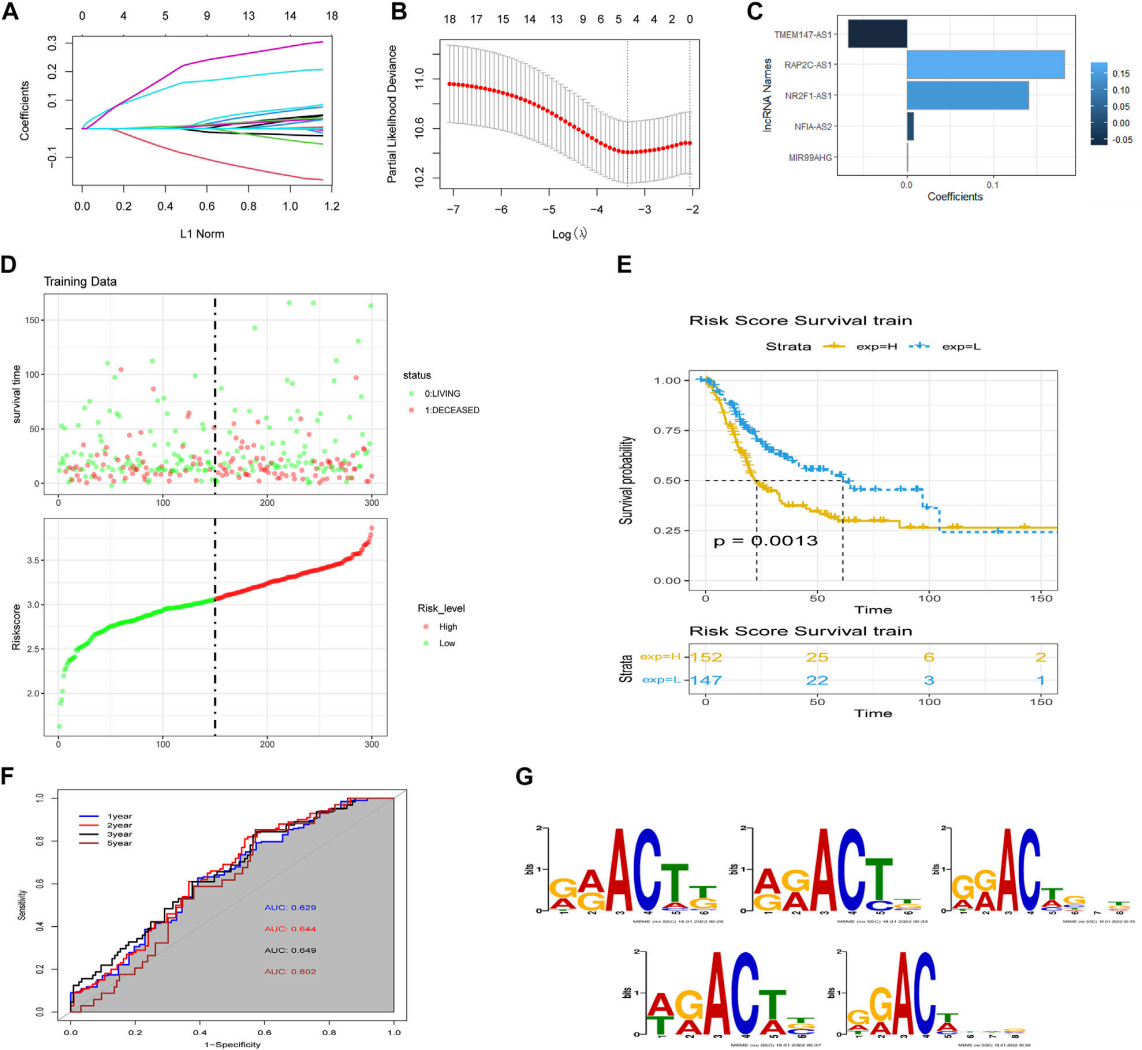
Construction of the m6A-lncRNA-associated prognostic risk scores (RSs). **(A)** least absolute shrinkage and selection operator (LASSO) regression was performed to calculate the minimum criteria and **(B)** 10-fold cross-validation for the optimal parameter selection in the LASSO Cox regression. **(C)** coefficients of each lncRNA. **(D)** distributions of RSs based on five m6A-lncRNAs and the survival status of BLCA patients from the TCGA dataset. **(E)** Kaplan–Meier curves showing that patients with different RS levels had different overall survival (OS) in the TCGA dataset. **(F)** time-dependent receiver operating characteristic (ROC) curves for predicting 1, 2, 3, and 5-year OS. **(G)** sequence logo depiction of the base content of potential m6A binding sites present on the five lncRNAs (generated using the MEME program). From top to bottom are MIR99AHG, NFIA-AS2, NR2F1-AS1, RAP2C-AS1, and TMEM147-AS1.

We performed LASSO Cox regression analysis on the 18 lncRNAs in the training set, which led to the selection of five m6A-lncRNAs with promising prognostic features (Figures 3A and B). RSs for patients with BLCA were calculated according to the formula: 0.0076 × NFIA-AS2 expression level) + (0.1409 × NR2F1-AS1 expression level) + (0.0011 × MIR99AHG expression level) + (−0.0678 × TMEM147-AS1 expression level) + (0.1822 × RAP2C-AS1) (Figure 3C). The median RS was used as a cut-off point for classifying BLCA patients into high- and low-risk groups. Risk profile plots show that patients in the high-risk group have significantly lower survival rates than those in the low-risk group (Figure 3D). Kaplan–Meier curves showed that a high RS predicted a poor prognosis for BLCA patients (*p* = 0.0013; Figure 3E), whereas the AUC was 0.649, demonstrating the ability of the RS to predict the prognosis of BLCA patients (Figure 3F). We obtained consistent results in the validation set (Supplementary Figure S1A–C). The m6A binding sites present in all five lncRNAs (*NFIA-AS2, NR2F1-AS1, MIR99AHG, TMEM147-AS1, and RAP2C-AS1*) were discovered using the RMBase v2.0 database (http://rna.sysu.edu.cn/rmbase/index.php). As shown in Figure 3G, sequence logos (generated using the MEME program) revealed the base content of the potential m6A binding site on three of the five lncRNAs.

### Evaluation of risk score as an independent prognostic factor for patients with bladder cancer

**FIGURE 4:**
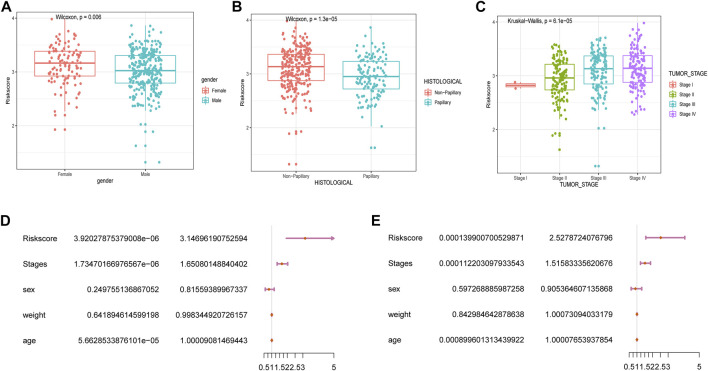
Correlation between RSs and clinicopathological features. Analysis of differences between RSs and clinicopathological features including **(A)** gender, **(B)** histological features, and **(C)** cancer stage. **(D)** univariate Cox and **(E)** multivariate Cox analysis of RSs and clinical variables.

We subsequently explored the relationship between RSs and clinicopathological characteristics. Correlation analysis showed that RSs were higher in women compared to men (Figure 4A). RSs also differed significantly by histologic subtype, with patients suffering from nonpapillary cancer having a worse prognosis than those with papillary disease (Figure 4B). The RS also correlated with cancer grading (Figure 4C).

Next, we performed univariate and multivariate Cox analyses to assess whether the selected five m6A-lncRNAs were independent prognostic factors for patients with BLCA. Results of the univariate Cox analysis indicated that an RS based on these five m6A-lncRNAs was significantly correlated with OS [hazard ratio (HR) = 3.15, *p* < 0.001] (Figure 4D). The multivariate Cox regression analysis revealed that the HR was 2.64 (*p* < 0.001; Figure 4E), indicating that an RS based on five m6A-lncRNAs could independently predict the prognosis of patients with BLCA.

### Construction of the nomograph

Nomograms were used to quantify the prognosis of patients with BLCA. To build a clinically applicable effective tool to predict the 1-year, 2-year, 3-year, and 5-year OS of patients with BLCA, we generated a nomogram, which included age, gender, cancer stage, weight, RS, and 1/2/3/5-year survival probability (Supplementary Figure S2A). Furthermore, the calibration plots indicated that the predicted vs. observed rates of 1-, 2-, 3-, and 5-year OS of patients with BLCA matched exactly (Supplementary Figure S2B). These data indicate that the RS was an independent prognostic factor for patients with BLCA and could inform future clinical prognosis evaluation.

### Analysis of tumor immune cell infiltration and the potential for immunotherapy

**FIGURE 5:**
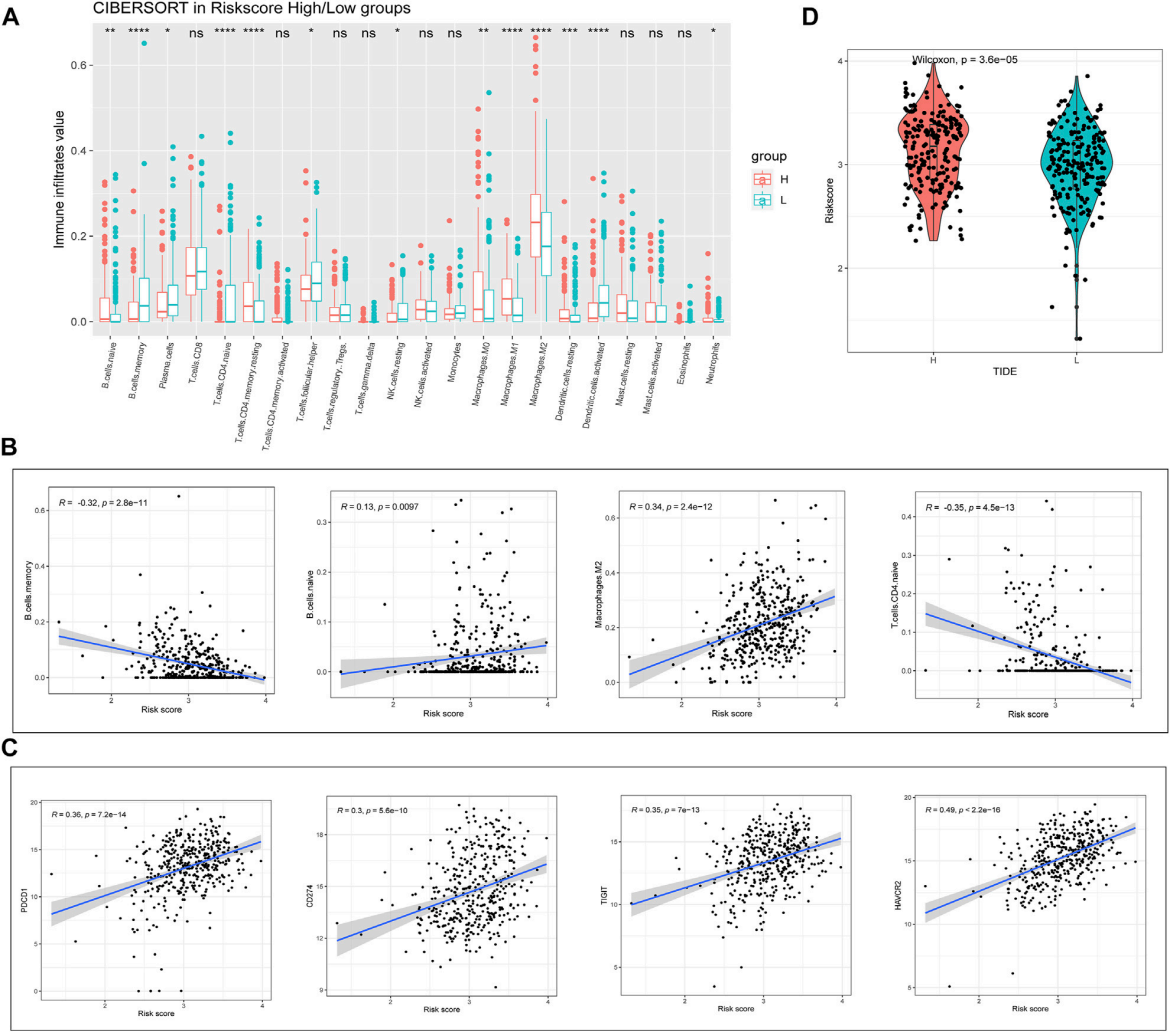
Evaluation of risk value and tumor immune infiltration. **(A)** analysis of the differences between high- and low-risk groups of patients with BLCA in relation to the expression of markers associated with 22 immune cell types. **(B)** correlation of immune cells with RSs. **(C)** correlation of immune checkpoints with RSs. **(D)** tumor immune dysfunction and exclusion prediction of immune escape ability in high- and low-risk groups of patients with BLCA.

We next proceeded to identify whether there was a difference in the tumor microenvironment (TME) between the two TCGA-BLCA cohort risk groups. CIBERSORT was performed to calculate the enrichment scores of 22 immune-related cells in each sample. Among patients with BLCA, the high-risk group frequently exhibited higher levels of immune cell infiltration (especially comprising naïve B-cells, memory CD4^+^ T-cells, resting macrophages, and resting dendritic cells (DCs)). However, the expression of markers associated with memory B-cell and activated DC infiltration was significantly downregulated in the high-risk group (*p* < 0.01; Figure 5A). Correlation analysis showed that the RS was significantly correlated with the expression of memory B-cell-, M2 macrophage-, and naïve CD4^+^ T-cell-associated markers (|R| > 0.3; *p* < 0.001; Figure 5B). Considering the clinical applications and benefits of immune checkpoint inhibitors, we also compared the correlation between RSs and common immune checkpoints. To our surprise, RSs showed a significant positive correlation with PD-1, PD-L1, TIGIT, and TIM3 levels (R > 0.3; *p* < 0.001; Figure 5C). We used the tumor immune dysfunction and exclusion (TIDE) algorithm to assess the likelihood of immune escape for the tumor samples. As shown in Figure 5D, tumor immune escape was stronger in high-risk patients with BLCA. The above results suggest that the degree of immune cell infiltration in BLCA differs from the RS predicted by m6A-lncRNAs, which may influence the effect of immunotherapy.

### Function and pathway enrichment analysis based on risk signatures

**FIGURE 6:**
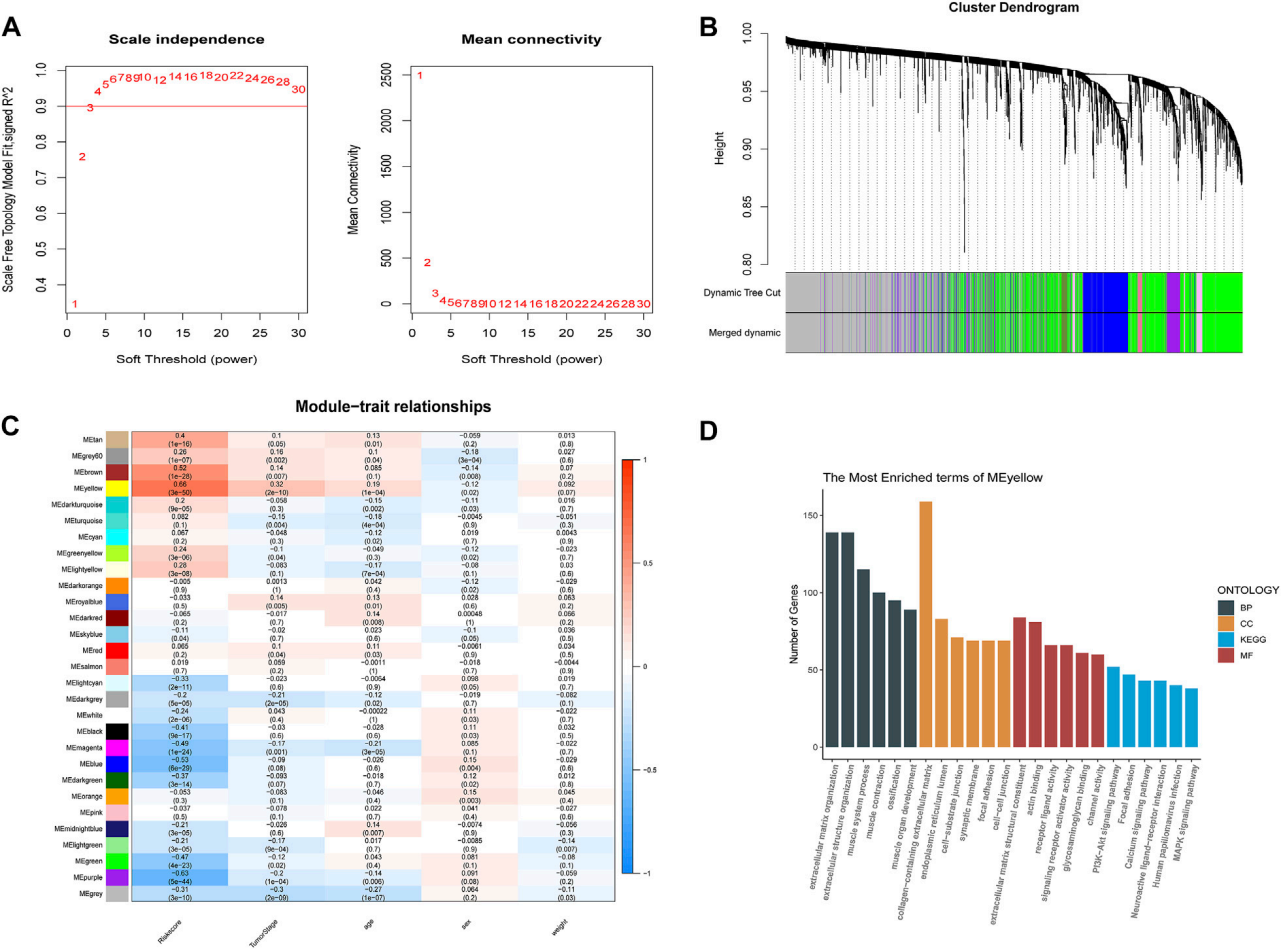
Weighted gene co-expression network analysis and functional enrichment analysis. **(A)** screening of the soft threshold. **(B)** clustering dendrogram of genes. **(C)** correlations between module feature genes and RSs and clinicopathological indicators. Each cell contains the corresponding correlation coefficient and *p*-value. **(D)** KEGG and GO analysis of yellow module genes.

To better understand the network interactions between risk models and other genes, we extracted clinical information including age, cancer grade, sex, and risk values for each sample used in the WGCNA analysis. The gene modules that correlated most strongly with the risk value were selected. When constructing a weighted gene network, the threshold of the adjacency matrix should satisfy the criterion that the network is close to being scale-free; as the threshold for network construction in our study, 3 was chosen (Figure 6A). The co-expression modules were then constructed, and similar modules were clustered to obtain a final set of 29 gene modules (Figure 6B). The results of the correlation analysis between gene modules and risk values showed that yellow modules displayed the highest degree of correlation with risk values (Cor = 0.66, P = 3e-50; Figure 6C). GO and KEGG enrichment analyses were then performed using genes identified from the yellow modules. We selected the top five terms for display. The GO analysis highlighted biological processes, cellular components, and molecular functions, mainly involved in extracellular matrix construction and muscle tissue development processes, with molecular functions related to actin binding, glycosaminoglycan binding, and receptor ligand activity. The results of the KEGG pathway enrichment analysis showed that the yellow module was mainly enriched in MAPK, PI3K-Akt, and calcium signaling pathways (Figure 6D), which are implicated in tumor progression.

**FIGURE 7:**
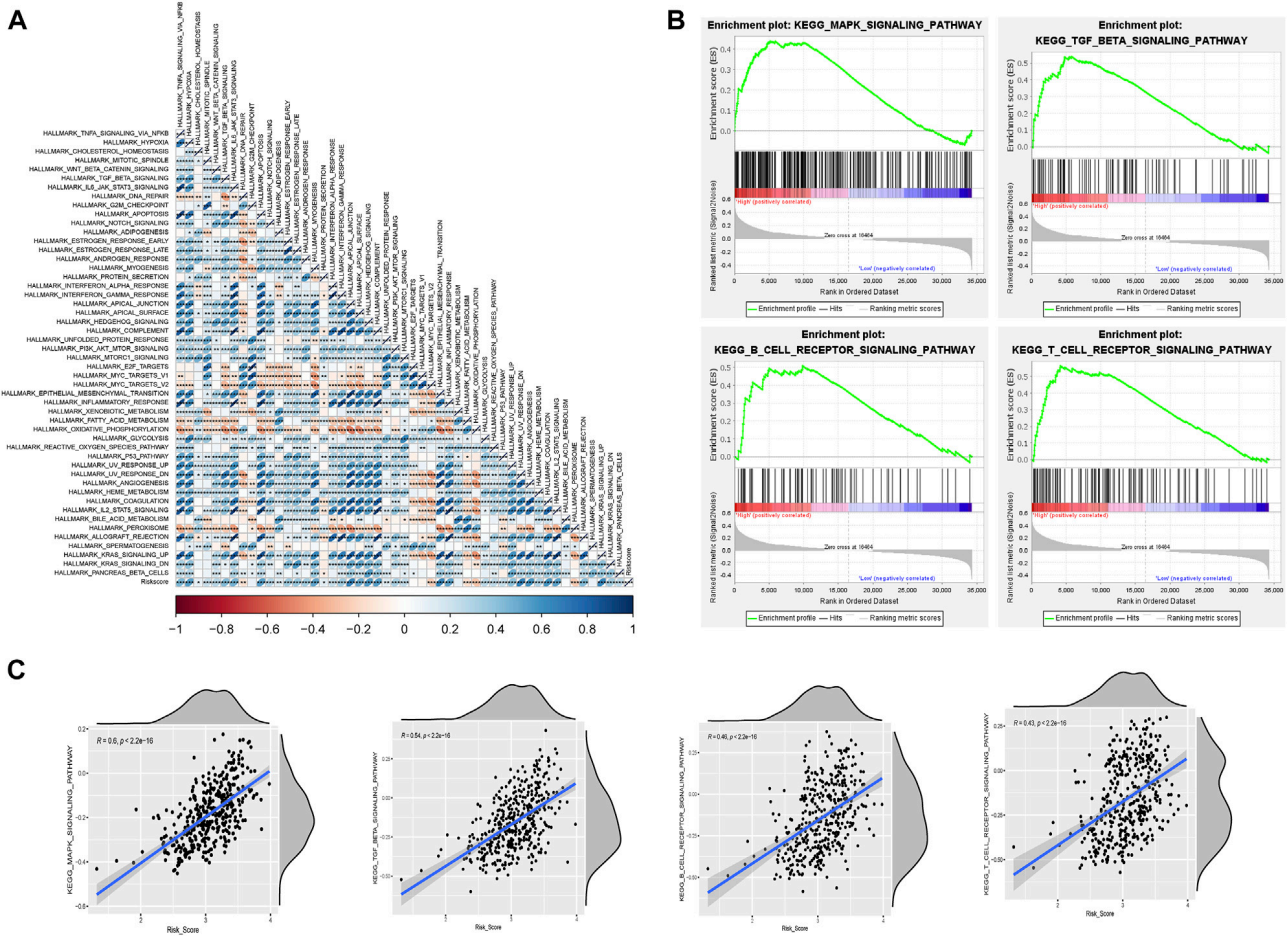
Risk model correlation path analysis. **(A)** correlation analysis of Hallmark gene sets with risk values based on the single-sample gene set enrichment analysis (ssGSEA) algorithm. **(B)** GSEA for assessing high- and low-risk differential pathways. **(C)** validation of the correlation between differential pathways based on the ssGSEA algorithm.

We then further explored the relationship between risk values and tumor characteristics. Correlation analysis, based on Hallmark gene set ssGSEA scoring, showed that risk values were associated with most cancer risk factors, especially apical junction, epithelial mesenchymal transition, and UV response DN (R > 0.4; *p* < 0.001; Figure 7A). In addition, GSEA analysis was used to compare different pathways between high-risk and low-risk groups based on m6A-lncRNA-derived prognostic signatures. As anticipated, the MAPK signaling pathway was also enriched, and we saw that some B-cell and T-cell response pathways were activated, which serves as additional proof that the risk signature we constructed is associated with immunity (Figure 7B). We verified this conclusion by performing further correlation analyses (Figure 7C).

### Assessment of the expression and prognostic value of the five m6A-lncRNAs selected

**FIGURE 8:**
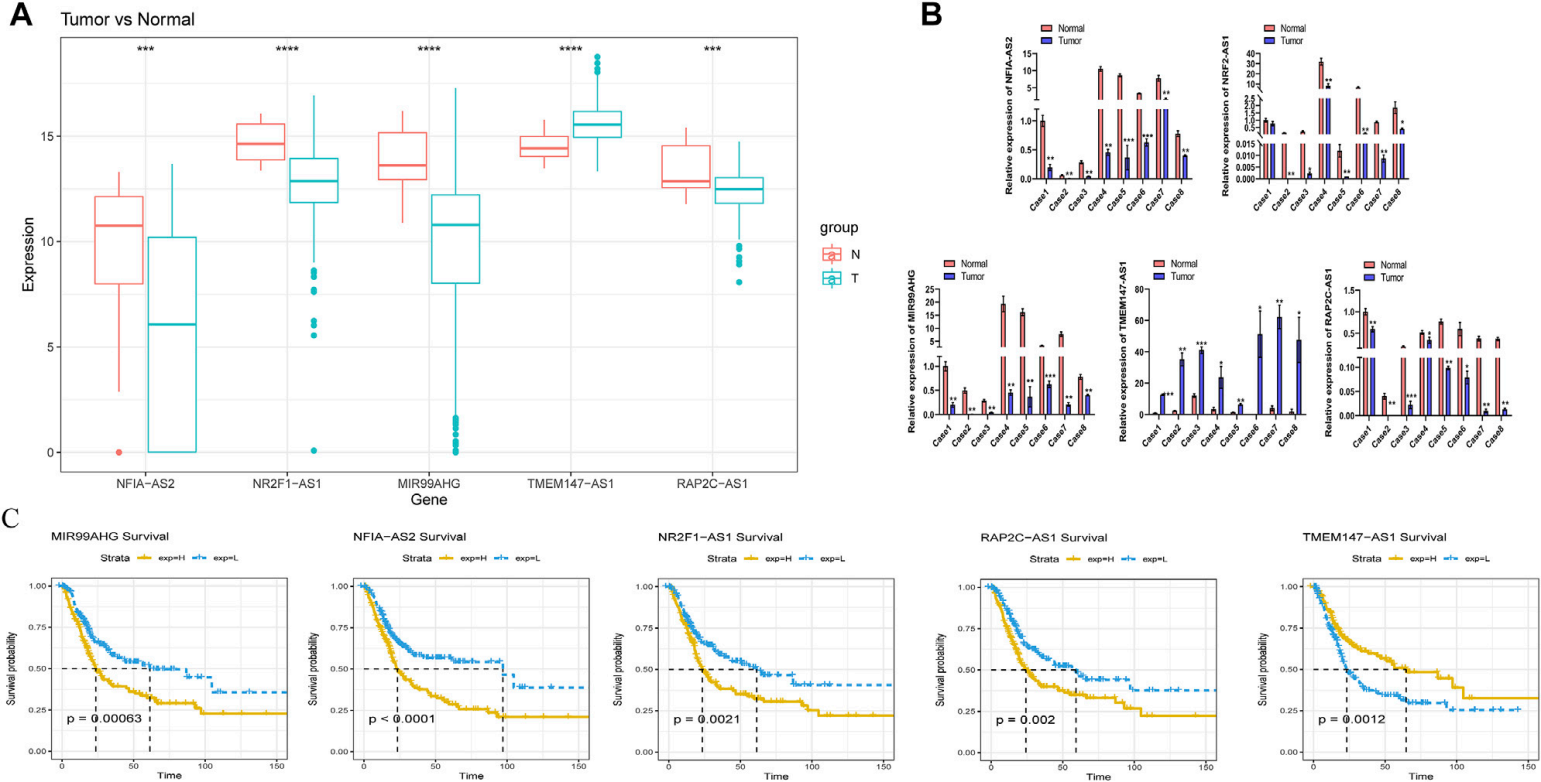
Evaluation of the prognostic and expression values of model genes. **(A)** expression of five m6A lncRNAs in BLCA patients. **(B)** the mRNA level of five m6A lncRNAs in 8 pairs of BLCA tissues and their paired normal adjacent tissues. **(C)** Kaplan–Meier survival curve of five m6A lncRNAs in BLCA patients.

We further explored the prognostic and expression values of the five selected m6A-lncRNAs in patients with BLCA. We analyzed whether there were differences in the expressions of these m6A-lncRNAs between normal and cancerous tissues. To our surprise, NFIA−AS2, NR2F1−AS1, and MIR99AHG were upregulated in tumors, while TMEM147−AS1 and RAP2C−AS1 were downregulated (Figure 8A). qRT-PCR was used to detect the expressions of these five lncRNAs in eight pairs of BLCA cases and adjacent healthy tissues. In most BLCA tissues, the results of pairwise difference analysis were consistent with those observed in our database (*p* = 0.05; Figure 8B). The Kaplan–Meier survival curve showed that the expression of all five selected m6A-lncRNAs was associated with the survival of patients with BLCA (all *p* < 0.05; Figure 8C). The high expression of m6A-lncRNA TMEM147-AS1 was associated with poor prognosis in patients with BLCA, while the expressions of NFIA-AS2, NR2F1-AS1, MIR99AHG, and RAP2C-AS1 presented a good prognosis.

## Discussion

m6A represents the most prevalent epigenetic modification of RNA found in eukaryotes. Specific methylation of RNA molecules regulates their structure and protein–RNA interactions, which may affect RNA metabolism, cell signaling, cell survival, and cell differentiation (Roundtree et al., 2017). Increasing evidence has supported the notion that the dysregulation of m6A methylation is closely associated with tumor progression (Ma et al., 2019; Liu T. et al., 2020). lncRNA expression is one of the most frequently encountered transcriptional changes in cancer (Du et al., 2013). Experimental evidence suggests that lncRNAs can play an important role in tumorigenesis (Schmitt and Chang, 2016); however, little is known about the role of m6A-lncRNAs in cancer. Here we report for the first time that m6A-lncRNAs can be used as diagnostic biomarkers and therapeutic targets in BLCA and are also associated with immune cell infiltration into the tumor.

In the present study, a total of 433 patients with BLCA (obtained from TCGA) were included to explore the prognostic significance of lncRNAs in BLCA. Eighteen m6A-lncRNAs were initially selected for model construction. The LASSO Cox algorithm identified five model genes and calculated risk values for each patient with BLCA. The Kaplan–Meier survival curves showed that all five m6A-lncRNAs and risk values had great predictive power for the OS of patients with BLDA. The ROC curves verified the accuracy of the model. We then analyzed the correlation between prognostic signature and various clinicopathological features. We found that risk values increased with cancer grade. Men tended to have higher RSs than women, which is interesting given that the incidence of BLCA in men is roughly three times higher than that in women (Torre et al., 2015). The differential expression of m6A-lncRNAs between men and women could constitute one of the reasons behind the pathogenesis of male BLCA. Furthermore, we performed a multivariate Cox regression analysis, combining other clinicopathological features and prognostic signatures, and demonstrated that the m6A-lncRNA prognostic signature was an independent predictor of survival in patients with BLCA.

A nomogram is a simple visualization used for cancer prognosis prediction (Zhou et al., 2021). The rationale for using nomograms as diagnostic and prognostic tools is that they effectively facilitate communication between doctors and patients, thus streamlining clinical visits (Kattan and Marasco, 2010) and helping physicians make accurate decisions regarding diagnosis and treatment (Balachandran et al., 2015). To construct the nomograph, we combined clinicopathological characteristics (including age, sex, weight, and cancer grade) with risk values. The calibration curves displayed a high degree of agreement between the predicted and actual 1-, 2-, 3-, and 5-year survival values, which will no doubt inform the clinical management of BLCA in the future.

The TME is an important component that contributes to tumor progression and metastasis (Lee et al., 2019). TME heterogeneity can affect multiple factors, including cancer prognosis and treatment response (Fridman et al., 2012). We found that high-risk patients with BLCA had higher levels of naïve B-cell- and M2 macrophage-associated markers. This is consistent with previous findings that M2 macrophages are often associated with poor prognosis in BLCA (Fridman et al., 2017). Checkpoint blockade immunotherapies are being extensively investigated in the treatment of various malignant tumors (Song et al., 2018). In our study, we were surprised to observe that the expressions of many immune checkpoint genes, including *PD-1* and *PD-L1*, showed a significant correlation between the two groups. In a similar way, the TIDE algorithm predicted that the immune evasion ability of the high-risk group was stronger than that of the low-risk group of patients with BLCA. The above results collectively imply that our model may be useful for predicting the response of patients with BLCA to immunotherapy.

In the present study, we identified five signature m6A-lncRNAs: *NFIA-AS2, NR2F1-AS1, MIR99AHG, RAP2C-AS1,* and *TMEM147-AS1*. Among them, lncRNA *NFIA-AS2* was previously shown to promote glioma progression by regulating the *miR-655-3p/ZFX* axis (Xin et al., 2020). Moreover, the lncRNA *NR2F1-AS1* was reported to be associated with the progression of various types of tumor, for instance by activating the IGF-1/IGF-1R/ERK pathway to promote breast cancer angiogenesis, inducing breast cancer lung metastasis dormancy by regulating NR2F1 and deltaNp63 (Zhang et al., 2020; Liu et al., 2021) and promoting the progression of papillary thyroid carcinoma (Yang et al., 2020) and esophageal squamous cell carcinoma (Zhang Y. et al., 2019). Han *et al.* suggested that *MIR99AHG* functions as a noncoding oncogene in lung adenocarcinoma (Han et al., 2021), but acts as an oncogene in acute megakaryocytic leukemia (Emmrich et al., 2014). *RAP2C-AS1* was previously reported to be associated with prognosis in esophageal cancer and renal clear cell carcinoma (Liu H. et al., 2020; Yang et al., 2021). We found that *RAP2C-AS1* was also closely associated with the prognosis of patients with BLCA, which warrants further mechanistic investigation. To date, *TMEM147-AS1* has only been reported once as a prognostic marker in BLCA (Zhong et al., 2021), which coincides with our results. Thus, our findings could help to identify the prognostic m6A-lncRNAs targeted by m6A regulators, thereby offering novel insights into the potential roles of m6A-lncRNAs in the tumorigenesis and progression of BLCA.

To explore the potential biological functions of the selected m6A-lncRNAs, we extracted the most relevant yellow module genes based on WGCNA and performed GO and KEGG analyses. The results indicated that the high-risk group of patients with BLCA was enriched in PI3K-Akt, MAPK, and calcium signaling pathways. Numerous studies have shown that the aberrant activation of the PI3K/Akt signaling pathway was critical for the tumorigenesis and progression of BLCA and could enhance the malignant phenotypes of this disease (Courtney et al., 2010). *In vitro* experiments using BLCA cell lines have shown that PI3K/Akt targets GSK-3β to regulate *ZEB1* transcription and promote tumor invasion (Wu et al., 2012). Therefore, the inhibition of PI3K–AKT signaling abolishes the invasiveness of BLCA cell lines (Wu et al., 2004). Wang *et al.* found that *NR2F1-AS1* is highly expressed in endometrial cancer (EC) and participates in the proliferation and migration of EC cells by regulating the PI3K/AKT/GSK-3β pathway. Our work suggests that high *NR2F1-AS1* expression correlated with PI3K–AKT signaling and is associated with poor prognosis in patients with BLCA. However, the exact mechanisms implicated require further elucidation.

In conclusion, we used a series of bioinformatic approaches to construct and validate an RS composed of five m6A-lncRNAs, which could have promising implications for predicting the prognosis of patients with BLCA. In addition, we generated a nomogram that could prove valuable in providing individualized treatment recommendations for BLCA.

## Data Availability

The original contributions presented in the study are included in the article/Supplementary Material; further inquiries can be directed to the corresponding authors.

## Abbreviations

BLCA, bladder cancer; m6A, N6-methyladenosine; TCGA, The Cancer Genome Atlas; RS, risk score; DEGs, differentially expressed genes; LASSO, least absolute shrinkage and selection operator; COX, the Cox proportional hazards model; OS, overall survival; GO, gene ontology; KEGG, Kyoto encyclopedia of genes and genomes; HR, hazard ratio; Coef, coefficient; DFS, disease-free survival; RFS, recurrence-free survival; DSS, disease-specific survival; PCA, principal component analysis; GSEA, gene set enrichment analysis; WGCNA, weighted gene co-expression network analysis; ssGSEA, single-sample gene set enrichment analysis; EC, endometrial cancer; TME, tumor microenvironment.
