# Supplementary figures and images for: Integrated risk scores from N6-methyladenosine-related lncRNAs are potential biomarkers for predicting the overall survival of bladder cancer patients
Source: Front Genet. 2022 Aug 17;13:906880. doi: 10.3389/fgene.2022.906880 (PMC9428265; doi:10.3389/fgene.2022.906880)

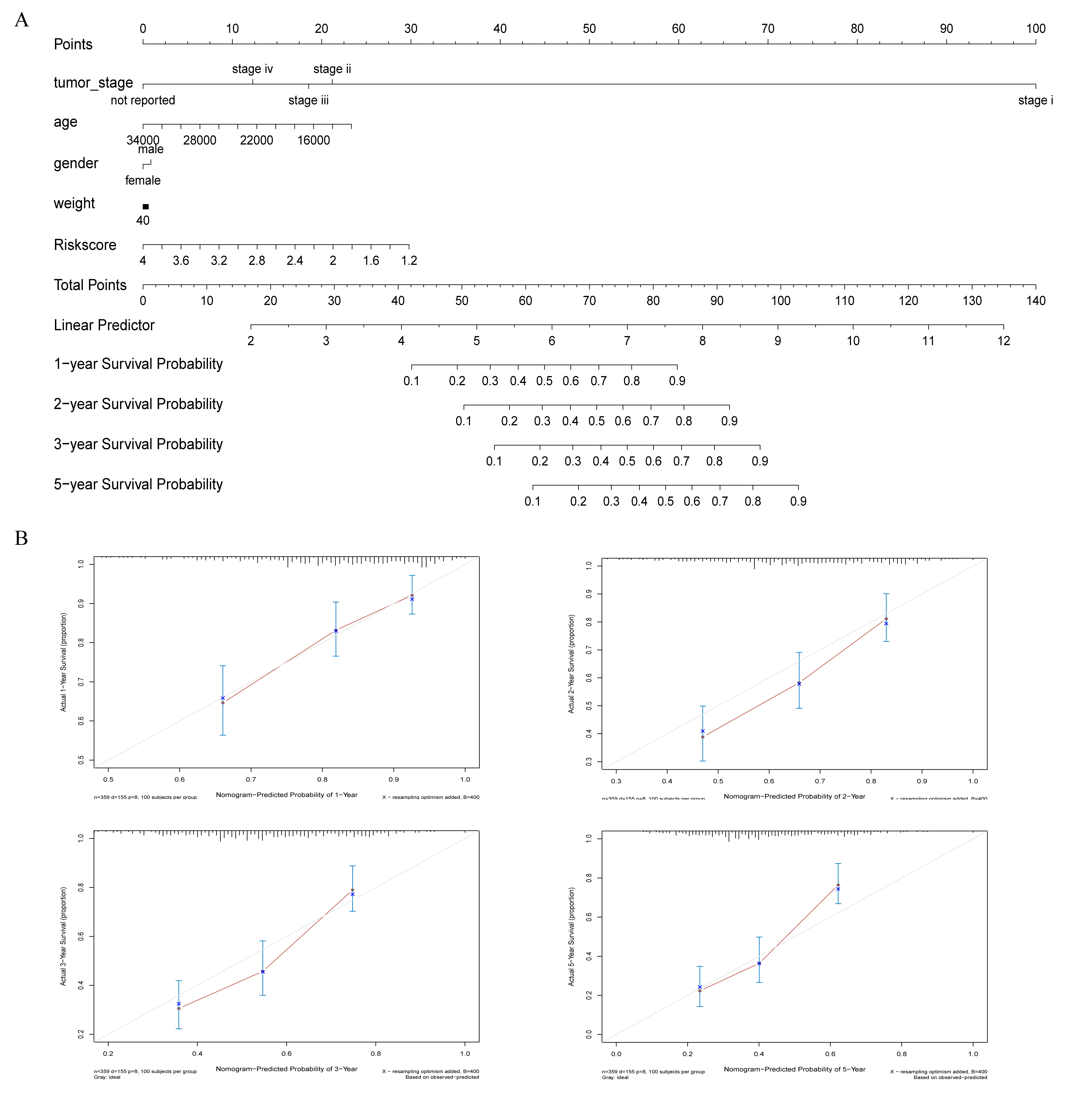

Supplement: Supplementary file 2 [file Image2.TIF]

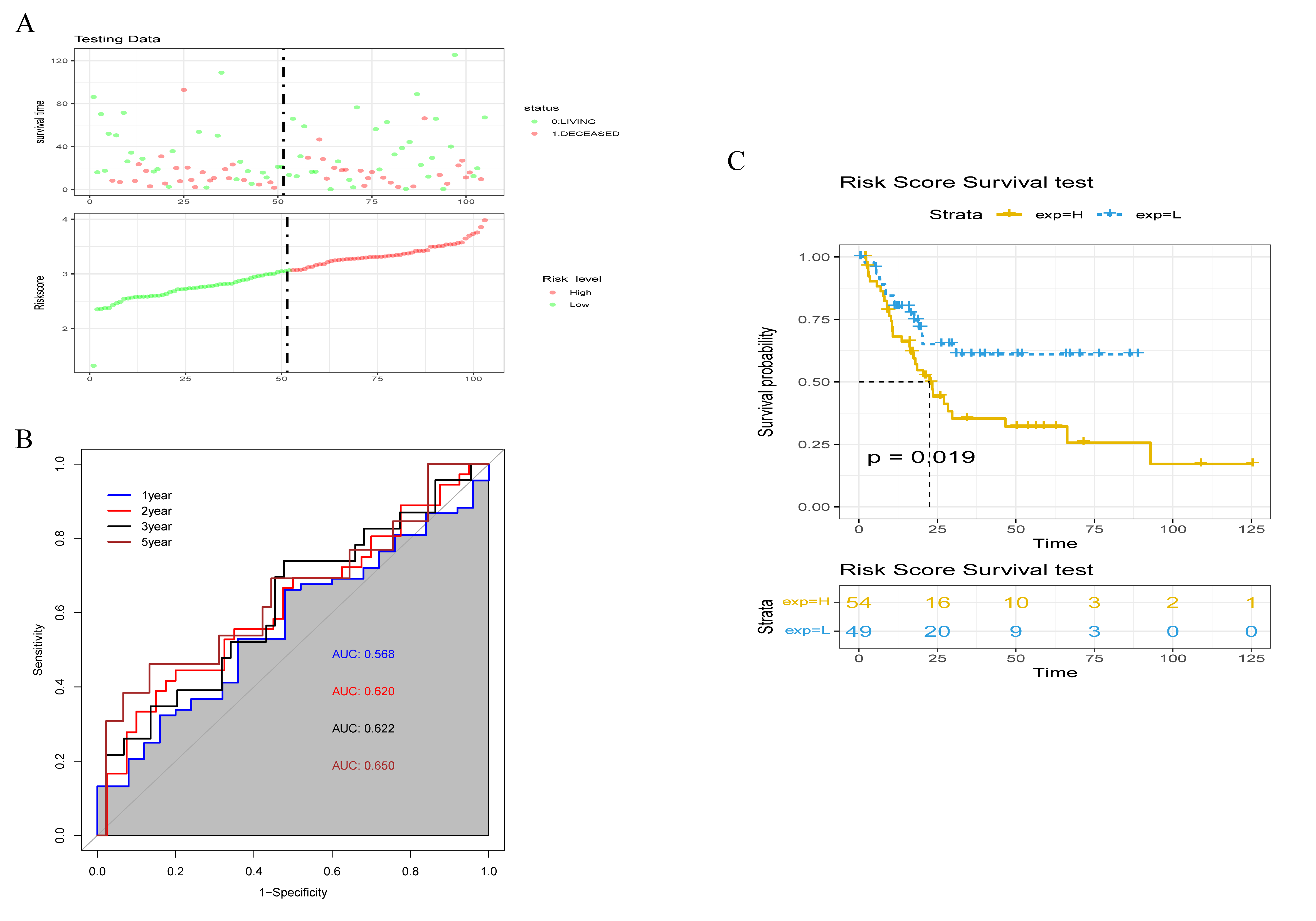

Supplement: Supplementary file 3 [file Image1.TIF]
